# TMEM16A is associated with voltage-gated calcium channels in mouse retina and its function is disrupted upon mutation of the auxiliary α_2_δ_4_ subunit

**DOI:** 10.3389/fncel.2015.00422

**Published:** 2015-10-21

**Authors:** Antonella Caputo, Ilaria Piano, Gian Carlo Demontis, Niccolò Bacchi, Simona Casarosa, Luca Della Santina, Claudia Gargini

**Affiliations:** ^1^Department of Pharmacy, University of PisaPisa, Italy; ^2^Centre for Integrative Biology, University of TrentoTrento, Italy

**Keywords:** retina, photoreceptors, TMEM16A, VGCC, *Cacna2d4*

## Abstract

Photoreceptors rely upon highly specialized synapses to efficiently transmit signals to multiple postsynaptic targets. Calcium influx in the presynaptic terminal is mediated by voltage-gated calcium channels (VGCC). This event triggers neurotransmitter release, but also gates calcium-activated chloride channels (TMEM), which in turn regulate VGCC activity. In order to investigate the relationship between VGCC and TMEM channels, we analyzed the retina of wild type (WT) and *Cacna2d4* mutant mice, in which the VGCC auxiliary α2δ4 subunit carries a nonsense mutation, disrupting the normal channel function. Synaptic terminals of mutant photoreceptors are disarranged and synaptic proteins as well as TMEM16A channels lose their characteristic localization. In parallel, calcium-activated chloride currents are impaired in rods, despite unaltered TMEM16A protein levels. Co-immunoprecipitation revealed the interaction between VGCC and TMEM16A channels in the retina. Heterologous expression of these channels in tsA-201 cells showed that TMEM16A associates with the CaV1.4 subunit, and the association persists upon expression of the mutant α2δ4 subunit. Collectively, our experiments show association between TMEM16A and the α1 subunit of VGCC. Close proximity of these channels allows optimal function of the photoreceptor synaptic terminal under physiological conditions, but also makes TMEM16A channels susceptible to changes occurring to calcium channels.

## Introduction

Photoreceptors are challenged to distribute their signal to a multitude of heterogeneous postsynaptic partners (Hoon et al., 2014). Rods feed mainly into rod bipolar cells and in smaller amount into cone bipolar cells (Dunn and Wong, 2012). Cones instead diverge their signal to multiple cone bipolar cell types at the level of their synaptic terminal (Wässle et al., 2009) and bipolar cells of at least 12 different types have been characterized in the mouse retina (Ghosh et al., 2004).

The efficiency of such a complex first synapse for the visual system relies upon the close proximity of the synaptic machinery components, as well as upon specialized synaptic structures called ribbons (Mercer and Thoreson, 2011; Schmitz, 2014).

Of particular importance for the correct development and function of photoreceptor synaptic terminals are the voltage-gated calcium channels (VGCC; Zabouri and Haverkamp, 2013), which are formed in mice by a complex of α_1_, β_2_ and α_2_δ_4_ subunits (Lee et al., 2015). Mutations in the α_1_ subunit of the channel are thus associated with disruption of the normal organization of the synaptic terminal and related to the insurgence of incomplete stationary night blindness in patients (Liu et al., 2013; Zabouri and Haverkamp, 2013).

The auxiliary subunits associated with α_1_ have been reported to play a role in regulating the functional properties of calcium channels and their correct trafficking to the synaptic terminal (Cantí et al., 2005; Felix et al., 2013). In particular, the localization of the α_2_δ_4_ subunit within photoreceptors is restricted to their terminals in the outer plexiform layer (OPL; De Sevilla Müller et al., 2013). Indeed, mutations of this subunit in *Cacna2d4* mutant mice have been reported to cause disarrangement of the synaptic terminal of photoreceptors (Wycisk et al., 2006a). We chose this mutant mouse model to investigate the fate of the VGCC/TMEM16A complex in the context of altered VGCC, without involving directly the α_1_ subunit.

VGCC channels mediate calcium influx in the synaptic terminal, initiating neurotransmitter release. Additionally, the local calcium influx is sensed by calcium-activated chloride channels, which in turn modulate neurotransmission. Amongst them, transmembrane member TMEM16A and TMEM16B channels are both expressed by rod and cone photoreceptors in mice (Stöhr et al., 2009; Billig et al., 2011; Jeon et al., 2013). Their expression is not conserved in rodents, since in rats TMEM16B is expressed only by rods (Dauner et al., 2013). Within photoreceptor synaptic terminals, TMEM16A localize diffusely (Mercer et al., 2011a; Jeon et al., 2013), while TMEM16B cluster at the level of ribbon structures (Stöhr et al., 2009) and are about 10 times less sensitive to calcium than TMEM16A (Vocke et al., 2013).

In addition to photoreceptor synaptic terminals, TMEM16A have been detected on retinal arterioles, where their disruption lowers blood pressure (Heinze et al., 2014).

Here, we report that VGCC and TMEM16A channels co-immunoprecipitate in wild type (WT) mice as well as in a heterologous system, placing chloride channels in proximity to local calcium influx domains. We find that in *Cacna2d4* mutant mice, the complex VGCC/TMEM16A is still present, but fails to reach the disorganized synaptic terminal. Our results expand the current view of the organization of photoreceptor synaptic terminals and suggest a link between the fate of VGCC and TMEM16A channels with disease conditions.

## Materials and Methods

### Animals

Mice (strain AJ/C57BL/10) were kept in a local facility with water and food *ad libitum* in a 12 h light/dark cycle with illumination level below 60 lux. In *Cacna2d4* mice, a mutation in exon 25 (c.2451insC) generates an early stop codon in exon 26, leading to a truncated α_2_δ_4_ subunit (Wycisk et al., 2006a). Mice were handled according to Italian laws and following the European Communities Council Directive of 24 November 1986 (86/609/EEC) for the use of animals in research, with experimental protocols approved by the Animal Care Committee of the University of Pisa, Italy. Animals were deeply anesthetized by an intraperitoneal injection of Urethane 20% in saline buffer (0.9% NaCl) at a dosage of 0.1 ml/10 g. At the end of the experiment mice were euthanized by cervical dislocation.

### Electroretinogram Recording (ERG)

The general procedure for animal preparation, anesthesia, ERG recording, light stimulation and data analysis have been previously described in detail (Della Santina et al., 2012; Piano et al., 2013). Briefly: ERGs were recorded in complete darkness via coiled gold electrodes making contact with the moist cornea. A small gold plate placed in the mouth served as both reference and ground. Responses were amplified differentially, band-pass filtered at 0.1–500 Hz, digitized at 12.8 kHz by a computer interface (LabView 7; National Instruments) and stored on disc for processing. Responses to flashes were averaged with an interstimulus interval ranging from 20 s for dim lights to 60 s for the brightest flashes. The full field illumination of the eyes was achieved via a Ganzfeld sphere 30 cm in diameter, whose interior surface was coated with a highly reflective white paint. Photopic ERG recordings were obtained by superimposing the test flashes on a steady background of saturating intensity for rods (30 cd/m^2^) after at least 10 min from the onset. ERG traces were analyzed using custom-compiled programs developed in LabView 7 (National Instruments).

### Immunohistochemistry

Retinas for immunohistochemistry were isolated and cryosections obtained as previously described (Della Santina et al., 2012). Cryosections (14 μm thick) were washed 3 times for 10 min in PBS and then incubated for 45 min in 1% bovine serum albumin (BSA) and 0.3% Triton-X100 in PBS to permeabilize membranes and block unspecific binding. Sections were incubated overnight at 4°C with primary antibodies (see Table 1), washed in PBS and then incubated for 2 h at room temperature in secondary antibodies (anti-mouse or anti-rabbit conjugated with Alexa Fluor 488, 1:500, Molecular Probes or with Rhodamin RedX, 1:1000, Jackson Immuno Research) diluted in 1% BSA in PBS. After washing in PBS they were cover slipped with Vectashield (Vector Laboratories). Retinal sections were visualized with a confocal microscope equipped with a krypton-argon laser (Leica TCS-SP5 Leica Microsystem, Germany); files were processed with image manipulation software (Photoshop CS2, Adobe Systems Incorporated, San Jose CA, USA).

**Table 1:** **List of antibodies**.

Antibody	Host	Dilution	Supply	Application
Protein Kinase C α	Mouse	1:100	Sigma Aldrich	IF
TMEM16A	Rabbit	1:100	Abcam	IF, WB, IP
TMEM16B	Rabbit	1:100	Abcam	IF
Pan α1	Rabbit	1:100	Sigma Aldrich	IF, WB, IP
Cacna2d4	Rabbit	1:400	Sigma Aldrich	IF, WB, IP
Cav1.4	Rabbit	1:100	Santa Cruz	WB, IP
Cav1.2	Rabbit	1:100	Santa Cruz	WB, IP
MPP4	Rabbit	1:200	Sigma Aldrich	IF
PSD95	Mouse	1:100	Millipore	IF, IP
Cone-Arrestin	Rabbit	1:5000	Millipore	IF
Syt2	Mouse	1:1000	ZIRC	IF
HCN1	Rabbit	1:100	Sigma Aldrich	IF
Calbindin	Rabbit	1:500	Swant	IF

*IF, Immunofluorescence; WB, Western blot; IP, Immunoprecipitation*.

### Cloning of Different Constructs

Cacna1f clone (encoding for murine Cav 1.4) was ordered from Source Bioscience (Nottingham, UK; GenBank: BC156573.1), amplified using primers Forward: CCACCATGTCGGAATCTGAAG, and Reverse: CCATGAGGCCTTAGAGGGC, and cloned into pIRES2-mCherry vector using SmaI restriction enzyme. Cacnb2 clone (encoding for murine β_2_ subunit) was ordered from Source Bioscience (Nottingham, UK; GenBank: BC115871), amplified using primers Forward: GCTAGATCTTTTTGCCGATGGTCC and Reverse: GACGGATCCTGCAGCTGTACTAG, and cloned into pIRES2-BFP vector using BamHI and BglII restriction enzymes. Ano1 clone (encoding for Tmem16a) was ordered from Source Bioscience (Nottingham, UK) in a pCMV6-Kan/Neo vector (GenBank: BC062959). Cacna2d4 WT and mutant (c.2451insC) clones (encoding for murine α_2_δ_4_ subunit) were described previously (Bacchi et al., 2015).

### Cell Culture and Transfection Conditions

tsA-201 cells (Cat. n.96121229 SIGMA) were maintained in DMEM supplemented with 10% FBS, 2 mM glutamine, 100 U/l Pen/Strep and grown at 37°C, 10% CO_2_. One day before transfection cells were split into 6 cm dishes (1 million cells/dish). Transfection was performed using Turbofect transfection reagent (Life Technologies; 6 μl for 2.5 μg DNA) with 0.5 μg Cacna1f, 0.5 μg Cacnb2, 0.5 μg TMEM16A, 0.5 μg Cacna2d4 WT or mutant vectors, and 0.5 μg pUC18 carrier DNA. Two days after transfection cells were washed and detached using PBS.

### Western Blot

TMEM16A and Calcium channel subunits were assessed by semi-quantitative western blot from *Cacna2d4* WT and mutant mice as well as in tsA-201 cell line. Proteins (100 μg) from either retinal or cell lysates were electrophoresed on 8% sodium dodecyl sulfate–polyacrylamide gel (SDS-PAGE). Proteins were transferred onto nitrocellulose membranes (0.45 μm, Sigma Aldrich) using a transfer buffer (25 mM Tris–HCl, pH 8.3, 192 mM glycine, 20% methanol). The protein blot was blocked by exposure to 5% non-fat dried milk and 0.1% Tween-20 (Biorad) in 20 mM Tris–HCl, 500 mM NaCl (Tris–buffered saline), pH 8, at room temperature, for 1 h. After the blocking procedure, the membrane was incubated overnight at 4°C with primary antibodies (see Table 1). The reactions were revealed by horseradish peroxidase-conjugated secondary antibody (anti-rabbit IgG, 1:10000, Millipore), incubated for 2 h at room temperature. Bands were visualized using a chemoluminescence kit (Immuno Cruz Western Blotting luminol reagent, Santa Cruz).

### Co-immunoprecipitation

Retinas from both WT and mutant mice (for each experiment, *n* = 4 animals were pooled) and the cell pellet were lysed with 200 μl of lysis buffer (50 mM Tris HCl pH 7.4, 150 mM NaCl, NP40 1%, Na-deoxycholate 0.25%, 1 mM EDTA, 2 mM PMSF, 2 mM sodium orthovanadate 0.1 M) supplemented with complete protease inhibitors cocktail (Sigma Aldrich). The lysis buffer was able to dissociate VGCC α_1_ subunit from α_2_ and δ chain, thus unmasking the epitope for the pan-α_1_ antibody. Lysates were then centrifuged at 10,000 g for 30 min at 4°C and the supernatants were collected. Protein concentrations were quantified used Bradford Reagent (Bio-Rad). For immunoprecipitation, 500 μg of total proteins obtained from retinal or cell lysate were incubated with 2 μg of primary antibodies overnight at 4°C. After pre-clearing, protein G-agarose beads (25 μl, Santa Cruz) were added to lysate and rotated at 4°C for 2 h. Beads were washed three times with Ripa buffer followed by centrifugation at 500 g for 5 min at 4°C. Immunoprecipitated proteins were eluted by boiling with sample buffer (1% SDS, 5% glycerol, 25 mM Tris-HCl, pH 6.8, 0.01% bromophenol blue, 0.5% 2-mercaptoethanol) and separated on 8% SDS-PAGE and then transferred onto PVDF membranes (porosity 0.45 μm, Millipore) for Western blot analysis. Proteins were immunodetected with anti-TMEM16A, anti-Panα1, anti-Cav1.2, anti-Cav1.4 and anti-Cacna2d4 antibodies (see Table 1); secondary antibodies were anti-rabbit HRP conjugated (Millipore 1:10000).

All antibodies were dissolved in 5% not dry fat -milk in TBS-Tween 0.1%.

Protein bands were visualized using a chemiluminescence kit (Immuno Cruz Western Blotting luminol reagent, Santa Cruz).

### Patch Clamp Recording of Mouse Photoreceptors

A retinal whole-mount isolated from light-adapted mice was oriented photoreceptor side-up and held in place by 80 μm-thick nylon threads glued to a stainless steel ring, while superfused with Locke’s saline (0.5 ml/min). A gigaseal was established using the blind patch approach (Blanton et al., 1989) and the access obtained using 270 μg/ml amphotericin B (Sigma-Aldrich, Italy), as previously reported for isolated mouse rods (Demontis et al., 2009). Intra and extracellular solution had similar osmolarity close to 300 mOsm/l. Rods were identified by their unique electrophysiological profile, i.e., the combination of an hyperpolarization-activated current (I_h_), a slowly-inactivating outward current (I_Kx_), a Ca^2+^ -dependent chloride current (I_Cl(Ca)_) and a small membrane capacitance (3–6 pF), as previously reported for isolated mouse rods (Demontis et al., 2009). I_h_ was activated by 2 s-long voltage steps to membrane potentials ranging from −50 to −120 mV, from a holding voltage of −40 mV. I_h_ was blocked by superfusion with Locke’s solution with added 3 mM CsCl. I_Cl(Ca)_ were activated by 2 s-long voltage steps ranging from −50 to −10 mV and inward tail currents generated by a 500 ms-long step to −70 mV. Currents were recorded with a HEKA EPC-8 amplifier, low-pass filtered at 0.2 KHz with a 3-pole Bessel Filter and digitized at 1 kHz by an Axodata 1200, using pClamp software 5.0 (Axon Instruments).

The impact of the *Cacna2d4* mutation on I_h_ and I_Cl(Ca)_ was assessed by comparing their fractional activation in rods of WT and MUT mice. Tail current amplitudes were measured at −70 mV. To remove the possible contribution from fast-deactivating calcium and potassium currents, for each rod and activating potential tail current amplitudes were averaged between 100 and 200 ms after imposing the voltage step. Tail current amplitudes were converted to conductance using Equation 1:

(1) $$G\left(V\right)\;=\;I_{\text{tail}}\text{/}(V-E_{x})$$

where *V* is the activating voltage preceding the step to −70 mV and *E*_x_ is the reversal potential of the current. *E*_x_ were −30 and −51 for I_h_ and I_Cl(Ca)_, respectively.

Voltage-dependent increases in conductance (*G(_V_)*) were fitted by Equation 2 using Origin 6.0 (Microcal):

(2) $$G(V)\;=\;\frac{G_{MAX}}{1+e^{(V-V_{0.5})/S}}$$

where *G_MAX_* is the maximal conductance, *V*_0.5_ is the half-activation voltage and *S* is the inverse slope factor.

## Results

### *Cacna2d4* Mutation Disrupts the Structure and Function of Photoreceptor Synaptic Terminals

**Figure 1:**
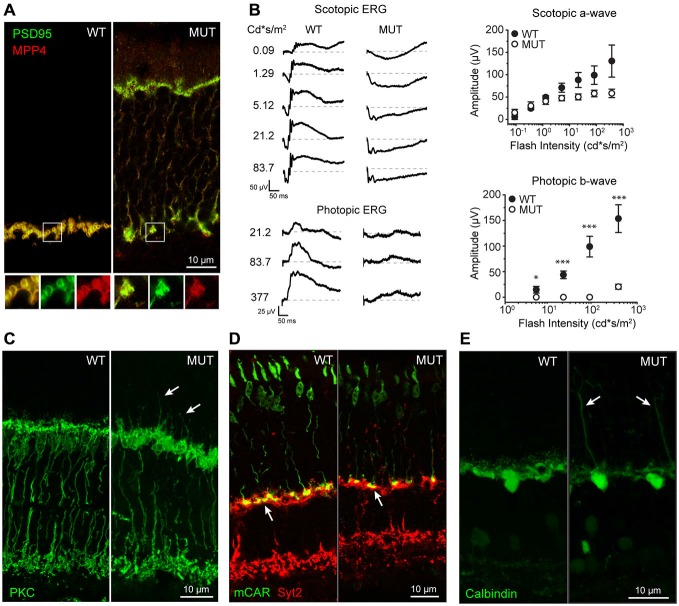
**(A)** Localization of presynaptic proteins MPP4 and PSD95 in wild type (WT) and *Cacna2d4* mutant mice (MUT). Bottom panels show magnification of the boxed regions: Merged signal (yellow), PSD95 (green), MPP4 (RED). **(B)** Scotopic and photopic ERG recordings at different flash intensity from WT and MUT mice. Dashed gray lines represent zero voltage level. Right plots: average amplitude of scotopic a-wave (top plot) and photopic b-wave (bottom plot) as a function of flash intensity. Values expressed as average ± SEM *n* = 4 animals for WT, *n* = 5 for MUT. Statistical comparison: *p < 0.05, ***p < 0.001 *t*-test. **(C)** Rod bipolar cell staining (PKC) in WT and MUT mice. Arrows indicate sites of dendritic sprouting of rod bipolar cells towards the outer nuclear layer (ONL). **(D)** Immunostaing of cones (MCAR, GREEN) and OFF cone bipolar cells (SYT2, RED) in WT and *Cacna2d4* mutant retinas. Arrows indicate the location of cone synaptic pedicles. **(E)** Calbindin staining of horizontal cells in WT and mutant mice. Arrows indicate sites of horizontal cell sprouting towards the ONL. Immunohistochemistry panels are representative of *n* = 6 mice for WT and MUT, respectively.

The normal arrangement of photoreceptor terminals involves the precise localization of synaptic proteins such as PSD95 and MPP4, with characteristic horseshoe shape, as shown in Figure 1A for WT. This highly specialized spatial arrangement is lost following mutation of the α_2_δ_4_ subunit of the VGCC in *Cacna2d4* mice. This leads to a disorganized photoreceptor synapse with synaptic proteins delocalized from the synaptic terminal towards the outer nuclear layer (ONL; Figure 1A). As a consequence *Cacna2d4* mutant mice fail to transmit the photoreceptor signals downstream, as indicated by the loss of b-wave in ERG recordings (Figure 1B). Despite these defects, photoreceptors are still able to respond to light, as indicated by presence of a-wave in the scotopic ERG recordings (Figure 1B), as also reported by Wycisk et al. (2006a). Average amplitude of scotopic a-wave (Figure 1B, right top plot) in *Cacna2d4* mutant mice is not significantly different from WT mice (*p* = 0.6, ANOVA 1-way). Additionally, we show that *Cacna2d4* mutant mice with the mixed C57BL/AJ background preserve a residual photopic ERG response although significantly reduced compared to WT mice (Figure 1B, right bottom panel), at variance respect to humans (Wycisk et al., 2006b) and mutant mice of the original C57BL/10 strain (Wycisk et al., 2006a).

We then investigated the possibility of postsynaptic rearrangements in *Cacna2d4* mutant mice. Rod bipolar cells (RBC) maintain their integrity and correctly stratify within the inner plexiform layer (IPL; Figure 1C). The lack of synaptic transmission from photoreceptors is associated with a diffuse sprouting of RBC dendrites outside of the plexiform layer into the photoreceptor’s nuclear layer (Figure 1C, arrows). Similarly to RBCs, also horizontal cell neurites ectopically sprout into the ONL (Figure 1E). OFF Cone bipolar cells are also present and their axons still laminate within the IPL (Figure 1D). Cone pedicles correctly target OPL in mutant mice (Figure 1D, arrows).

The OPL is therefore disrupted both morphologically and functionally in *Cacna2d4* mutant mice, with alterations observed in the axons of photoreceptors and in dendrites of their postsynaptic partners.

### TMEM16A Channels Lose their Localization and Function in *Cacna2d4* Mutants

**Figure 2:**
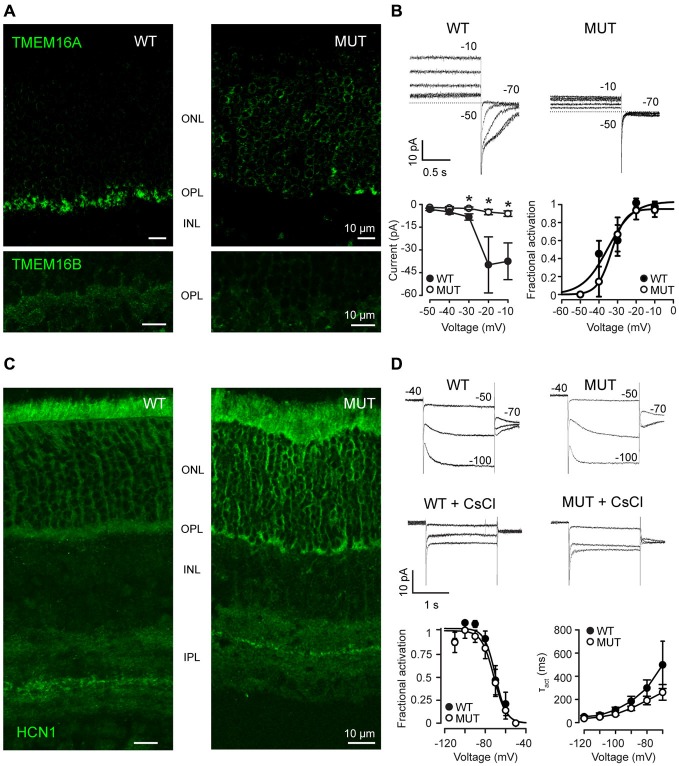
**(A)** Immunostaining of WT and mutant *Cacna2d4* (MUT) retinas for TMEM16A (upper panel) and TMEM16B (bottom panel) channels. **(B)** Perforated-patch clamp recordings show steady-state currents evoked in representative photoreceptors of *Cacna2d4* WT and mutant mice by voltages ranging from −50 to −10 mV, and the tail currents measured upon stepping to −70 mV. Numbers close to current traces report the applied voltages. Dashed lines plot the 0-current level. Note that at early times after establishing perforated-patch recordings, I_Cl(Ca)_ currents in response to voltage steps positive to −30 mV are outward. The currents, however, will turn inward at later recording times (not shown), as E_Cl_ shifts from about −50 to near 0 mV, due to the permeability of amphotericin pores to chloride and the use of 140 mM KCl in the pseudo-intracellular solution. The slow shift in E_Cl_ is consistent with the time required for the diffusion of chloride ions from the cell body compartment to the synaptic terminal. Circles in the left lower panel plot average tail current amplitudes (±SEM) at −70 mV (see “Materials and Methods” section). Circles in the right lower panel plot average values of the normalized fractional activation of g_Cl(Ca)_ (the conductance carrying I_Cl(Ca)_), generated by averaging data from individual fits in 8 WT and 5 MUT rods. Curves plot best fits to average data by Equation 2 (see “Materials and Methods” section). Fractional activation was estimated in 5 out of 6 MUT rods, because g_Cl(Ca)_ in the remaining rod was too small for curve fitting. Average values (±SEM) of the maximal conductance resulting from individual fits were 1.02 ± 0.45 pS (WT, *N* = 8) and 0.14 ± 0.06 (MUT, *N* = 5). The difference between WT and MUT rods was statistically significant (*p < 0.05 by Mann-Whitney U-test). Average *S* values from individual fits were smaller than those generated by fits to average data due to the spread in *V*_0.5_ values between individual rods. **(C)** Immunostaining for Hcn1 channels in *Cacna2d4* WT and mutant retinas. **(D)** Patch clamp recording of I_h_ in *Cacna2d4* WT and mutant photoreceptors. Application of 3 mM CsCl to the perfusion bath confirmed the presence of I_h_ currents. Numbers close to current traces indicate the applied voltages. Circles in the lower leftmost panel plot average values (±SEM) of the fractional activation of the conductance (g_h_) carrying I_h_, computed from individual fits in 8 WT and 6 MUT rods. Curves are best fits to data of Equation 2 (see “Materials and Methods” section). Circles in the lower rightmost panel plot the average time constants (τ_act_) of I_h_ activation in the range −70/−120 mV. Time constant were estimated using Clampfit 8.0 to best fit currents with a single exponential function of the form: *I(t)* = *Ae^−t/τ_act_^* where *A* is the amplitude of the activated current and *τ*_act_ is the time constant of activation.

In WT mice, the chloride channel TMEM16A is normally localized in photoreceptors, particularly in their synaptic terminals (Figure 2A), where the calcium influx occurs through VGCC. In mice mutant for the α_2_δ_4_ subunit of the calcium channel, TMEM16A proteins are no more present in the synaptic terminal of photoreceptors in the OPL but they are delocalized at the level of the cell body within the ONL (Figure 2A, top panels). At the same time, also TMEM16B channels lose their localization at the level of OPL in mutant mice (Figure 2A, bottom panels). To assess the functional relevance of displaced TMEM proteins, we measured Ca^2+^ -dependent chloride current (I_Cl(Ca)_). In WT rods recorded in Locke’s solution (Figure 2B), voltage steps positive to −50 mV activates outward currents, which turn inward upon stepping at −70 mV as expected from the chloride reversal potential of about −50 mV (see Figure 2). These currents are Ca^2+^-dependent, as they are strongly reduced by the substitution of Ca^2+^ with Ba^2+^ (not shown). Furthermore, the decay of inward tail currents at −70 mV in response to depolarization to −20 and −10 mV occurs over a time-span longer than 500 ms, which is characteristic of I_Cl(Ca)_ and is set by the kinetics of Ca^2+^ clearing from the synaptic terminal (Morgans et al., 1998). Overall, these properties are consistent with Ca^2+^-dependent chloride-selective channels of WT mammalian rods (Cia et al., 2005). On the other hand, I_Cl(Ca)_ are nearly abolished in photoreceptors of *Cacna2d4* mice, as shown by the lack of both outward currents in the range −40/−10 mV and large inward tail currents at −70 mV. Similar voltage-gated currents are recorded from mutated rods (MUT) and enzymatically-dissociated rods lacking their synaptic terminals (Demontis et al., 2009), an observation consistent with the localization of I_Cl(Ca)_ to the synaptic terminal, as reported for salamander rods (MacLeish and Nurse, 2007). Average values (±SEM) of inward tail current amplitudes at −70 mV are plotted as a function of activating voltages in the lower panel for WT (filled circles, *N* = 8) and MUT (open circles, *N* = 6) rods. The average amplitude (±SEM) of the tail current in WT reaches a maximum value of −39.4 ± 18.5 pA at −20 mV, matching the peak inward calcium current reported for WT mouse rods (Morgans et al., 2005). The average tail current amplitude at −20 mV in MUT rods was −5.0 ± 1.8 pA, and the difference between WT and MUT rods was statistically significant (*p < 0.05 by Mann-Whitney U-test). Significant differences were also found for tail current amplitudes at −10 and −30 mV (*p < 0.05 by Mann-Withney U-test). The loss of I_Cl(Ca)_ may indicate a defect in TMEM16 channels trafficking to the plasma membrane and/or a reduced activation secondary to a decrease in Ca^2+^ influx through VGCC. To analyze in more detail the effect of the *Cacna2d4* mutation on I_Cl(Ca)_, we compared its voltage-dependent activation in WT and MUT rods. A plot of the average normalized fractional activation of I_Cl(Ca)_ conductance (see “Materials and Methods” section) is shown in the lower right panel of Figure 2B for WT and MUT rods. The average (±SEM) half-activation voltages computed from individual fits were −35.9 ± 3.1 mV and −33.3 ± 3.4 mV in WT (*N* = 8) and MUT (*N* = 5) rods, respectively. The difference was not statistically significant (*p* = 0.42 by Mann-Whitney U-test). The average values of *S* were 2.3 ± 0.3 mV and 2.6 ± 0.3 mV in WT and MUT rods, respectively, and the difference was not significant (*p* = 0.41 by Mann-Whitney U-test). The similar voltage-dependence of activation in WT and MUT rods indicates that the mutation impairs TMEM16 channels trafficking to the plasma membrane, but does not affect the functional coupling between TMEM16 and VGCC.

To assess whether the loss of I_Cl(Ca)_ represents an unspecific effect of the *Cacna2d4* mutation on ion channels trafficking to the plasma membrane, we analyzed the distribution and the functional properties of voltage-gated channels with localization in other portions of photoreceptors, such as the isoform 1 of hyperpolarization-activated cyclic nucleotide modulated channels (Hcn1), that carry I_h_. Hcn1 channels are still localized at the inner segment of photoreceptors and at the ONL in both WT and mutant mice (Figure 2C). At the functional level, I_h_ can be recorded in both WT and mutant rods and blocked by 3 mM CsCl (Figure 2D). Average normalized fractional activation curves are shown in the lower leftmost part of Figure 2D. Fractional activation parameters of the conductance underlying I_h_ indicate average (±SEM) half-activation voltages of −68.2 ± 2.7 mV and −71.8 ± 3.5 mV in WT (*N* = 8) and MUT (*N* = 6) rods, respectively. This difference is not statistically significant (*p* = 0.56, Mann-Whitney U-test). The average maximal conductance were 0.07 ± 0.01 nS and 0.11 ± 0.02 nS for WT and MUT rods, and the difference was not statistically significant (*p* = 0.40 by Mann-Whitney U-test). Circles in the lower rightmost panel plot average values of the time constant describing I_h_ activation kinetics (τ_act_) in 8 WT and 6 MUT rods, estimated by single exponential fits to current. Average values of τ_act_ at −110 mV (g_h_ is fully activated) are 64.3 ± 19.7 ms and 48.2 ± 7.9 ms for WT and MUT rods, respectively, and the difference is not statistically significant (*p* = 0.38 by Mann-Whitney U-test). At −70 mV, close to the half-activation voltage, τ_act_ are 498.3 ± 203.9 and 261.2 ± 67.6 ms for WT and MUT rods, respectively, and the difference is not significant (*p* = 0.52 by Mann-Whitney U-test). These results indicate that the mutation does not cause an unspecific impairment of ion channels trafficking to the plasma membrane.

Therefore, the reorganization of the synaptic terminal in photoreceptors of *Cacna2d4* mutant mice does not impair the function of the entire cell, but affects the trafficking of proteins normally localized in the synaptic terminal.

### TMEM16A Channels are Associated with the α_1_ Subunit of Calcium Channels

Our results show that a defect in VGCC accessory subunits leads to rearrangements of the photoreceptor synaptic terminal also involving channels that are not the primary target of the mutation, such as TMEM16A.

We further investigated the relationship between the mutation in *Cacna2d4* and affected proteins in heterologous expression system as well as in the retinal tissue.

**Figure 3:**
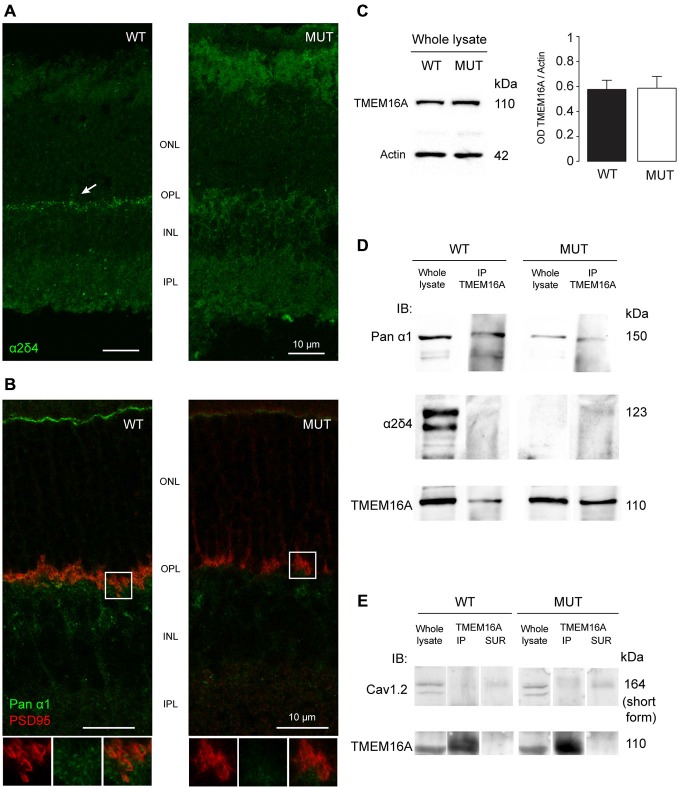
**(A)** Staining of α_2_δ_4_ subunit in WT and mutant (MUT) animals. The arrow in the WT images shows the punctate labeling for α_2_δ_4_ subunit in the outer plexiform layer (OPL). Puncta are absent in mutant retina. **(B)** Co-localization of the α_1_ subunit of calcium channels (Pan-α_1_, green) and photoreceptor ribbon (PSD95, red) in WT retina. In mutant retina the structure of presynaptic terminal is disorganized as shown by the lack of horseshoe-shaped PSD95 staining. Bottom images show magnification of the boxed area. **(C)** Western blot analysis of TMEM16A content in retinas of WT and MUT animals. Left images: Example western blots. Right histograms: Optical density ratio TMEM16A/β-actin in WT and MUT mice. Values expressed as average ± SEM, *n* = 8 mice per condition, *p* = 0.94, *t*-test. **(D)** Immunoblot in retinal tissue from WT and MUT mice with antibodies against the α_1_ subunit (top row) and the α_2_δ_4_ subunit (middle row) of calcium channels, either in whole lysate (left) or after co-immunoprecipitation of TMEM16A (right). The bottom row shows the control labeling against TMEM16A (input). Representative example of *N* = 3 independent Co-IP experiments. Each experiment is the measure obtained from retinas of *n* = 4 animals. **(E)** Immunoblot in retinal tissue from WT and MUT mice with the antibody against Cav1.2 subunit of calcium channel (top row) either in whole lysate (left lanes), after co-immunoprecipitation of TMEM16A (middle lanes) or in control supernatant from co-immunoprecipitation (right lanes). The control labeling against TMEM16A (input) is shown in the bottom panels. Representative example of *N* = 3 independent Co-IP experiments. Each experiment is the measure obtained from retinas of *n* = 4 animals.

In WT mice the localization of the α_2_δ_4_ subunit is at the level of OPL with a punctate distribution (Figure 3A, left panel). This specific localization at the level of photoreceptor synapses is lost in the *Cacna2d4* mutant mice (Figure 3A, right panel).

The localization of the main α_1_ subunit of VGCC has a characteristic punctate distribution at the level of the photoreceptor pedicles in association with PSD95 proteins (Figure 3B, left panel). In *Cacna2d4* mice, PSD95 and α_1_ subunit lose the characteristic shape at the level of the pedicles (Figure 3B, right panel). This rearrangement is compatible with a critical role of the α_2_δ_4_ subunit in the trafficking of VGCC towards the synaptic terminal.

Protein expression levels of TMEM16A in WT and mutant retinas are not statistically different (Figure 3C, *p* = 0.94, *t*-test), indicating that TMEM16A undergoes a process of delocalization within mutant photoreceptors rather than a reduced production or increased protein turnover.

Co-immunoprecipitation of TMEM16A with the α_1_ subunit of the calcium channel suggests that the two proteins interact in WT mice (Figure 3D). The interaction persists in *Cacna2d4* mutant mice despite both channels lose their specific localization. Although interacting with VGCC present in retinal neurons, we did not detect any interaction between TMEM16A and Ca_V_ 1.2 subunit (Figure 3E), specific of calcium channels present in the smooth muscle of blood vessels (Catterall et al., 2005; Narayanan et al., 2010), at the same time, TMEM16A did not label blood vessels at the level of OPL (Figure 2A).

### TMEM16A Belong to the Same Signaling Complex of VGCC via Interaction with Ca_V_1.4

**Figure 4:**
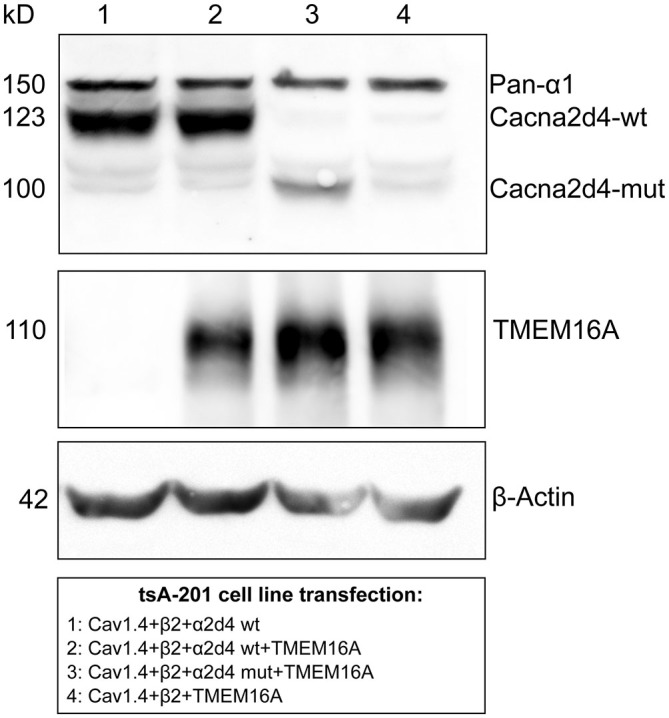
**Co-expression of mouse TMEM16A together with WT or mutant (MUT) α_2_δ_4_ variants transfected in tsA-201 cells.** A specific band for α2δ4 is visible following tsA-201 co-transfection with Cav1.4, β_2_, and constructs encoding either WT (lanes 1 and 2) or MUT (lane 3) α_2_δ_4_ variants. The α_2_δ_4_ band changes in size accordingly to the relative length of the expressed variant. In the negative control (lane 4) α_2_δ_4_ is not detectable, proving the absence of endogenous α_2_δ_4_ in tsA-201 cells. In the same cells, a specific band of TMEM16A is present after channel transfection (lane 2–4). Representative example of *N* = 3 experiments.

To test whether there is a interaction between TMEM16A and VGCC or via other proteins, we co-expressed these two channels in a heterologous system. In order for the calcium channel to be properly trafficked to the cellular membrane, transient expression of Ca_V_1.4, β_2_ and α_2_δ_4_ subunits was performed in tsA-201 cells, together with murine TMEM16A (Bacchi et al., 2015). Expression of transfected proteins was revealed by Western blot in Figure 4. WT or mutant form of α_2_δ_4_ subunit was expressed by the heterologous system (Figure 4, lane 2 and 3, respectively). Transfection experiments lacking the α_2_δ_4_ subunit or TMEM16A were provided as negative control (Figure 4, lane 1 and 4, respectively).

**Figure 5:**
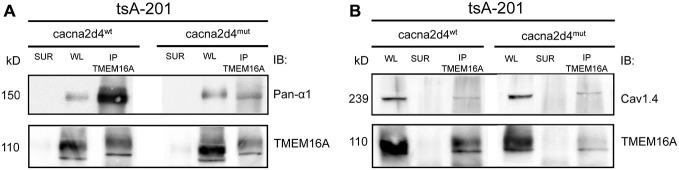
**Co-immunoprecipitation of TMEM16A (input) with the *α*_1_ subunit of calcium channels revealed by either an anti-Pan*α*1 (output, A) or anti-Cav1.4 (output, B) antibodies.** The tsA-201 cell line was co-transfected with TMEM16A, Cav1.4, β_2_, and constructs encoding either WT (*Cacna2d4^wt^*, left panels) or mutant (*Cacna2d4*^mut^, right panels) α_2_δ_4_ variants. WL, whole lysate; SUR, supernatant; IP, immunoprecipitate. Representative example of *N* = 3 experiments.

We then immunoprecipitated TMEM16A and revealed the co-immunoprecipitation of Ca_V_1.4 with either pan- α_1_ (Figure 5A) or Ca_V_1.4 (Figure 5B) antibodies, using buffer conditions where the multiple VGCC subunits are dissociated (see Material and Methods). Interaction between TMEM16A and CaV1.4 persists also in cells expressing the mutant α_2_δ_4_ subunit (Figures 5A,B, right lanes). Altogether our experiments indicate that TMEM16A and Ca_V_ 1.4 belong to the same signaling complex in WT mice as well as in mice with defects in the VGCC channel subunit α_2_δ_4_.

## Discussion

Results reported in this paper as well as previous literature (Ruether et al., 2000; Wycisk et al., 2006a) show that a mutation in an auxiliary VGCC subunit is sufficient to disrupt the normal architecture of the synaptic terminal of photoreceptors. The main traits of the alterations observed in *Cacna2d4* mutant mice resemble those described for mutations of the α_1_ subunit of the channel (Michalakis et al., 2014). Structural proteins of the synapse are delocalized towards the cell body compartment. Thus the ability of rod photoreceptors to signal downstream is severely impaired as indicated by the absence of b-wave in the scotopic ERG. Postsynaptic rearrangements are common to mice with transmission defects in photoreceptors. Bipolar and horizontal cells do not die but outgrow their dendrites in search of residually functional partners or, as described for α_1_ subunit mutants form ectopic synapses at the level of the photoreceptor cell body (Zabouri and Haverkamp, 2013).

Despite a major reorganization of dendrites in the OPL of *Cacna2d4* mutant mice, stratification of both rod bipolar and OFF cone bipolar cells axons is not appreciably altered. Resilience to alterations of the inner retinal circuitry compared to OPL has also been observed in other transmission defect mutants, such as *Cacna1f* mutants (Mansergh et al., 2005; Chang et al., 2006). It is possible that even a small residual activity of bipolar cells may be sufficient for the IPL circuit to maintain its structure as well as preservation of amacrine cell activity.

Our results show that TMEM16A channels are connected to the α_1_ subunit of calcium channels, resulting in advantages for the functionality of photoreceptor synaptic terminals. The close proximity of TMEM16A to calcium influx domains allows optimal feedback control of synaptic activity. At the same time, rearrangements of the position of calcium channels, as described in salamander photoreceptors (Mercer et al., 2011a,b) can also result in the repositioning of TMEM16A channels. The side effect of this interaction between the two channels is that alterations occurring to the calcium channel, such as those observed in *Cacna2d4* mutant mice, can easily disrupt TMEM16A localization. Indeed, both immunolocalization of TMEM16 proteins and patch-clamp recordings of I_Cl(Ca)_ currents indicate that the *Cacna2d4* mutation impairs TMEM16 channels trafficking to the synaptic compartment of rods. However, the similar voltage-dependence of I_Cl(Ca)_ tail currents in both WT and MUT rods indicates that the residual TMEM16 channels reaching the plasma membrane in MUT rods are still functionally coupled with VGCC.

The observation that I_Cl(Ca)_ has similar I/V in both WT and MUT rods may be surprising in view of the reduction in peak amplitudes of VGCC-mediated currents in tsA-201 cells co-expressing Cav1.4 with MUT *Cacna2d4* isoform compared to WT (Bacchi et al., 2015), similarly to mouse rods in *Cacna2d4* mice (unpublished observations). A reduced influx through VGCC in the presence of a maintained extrusion by the Ca^2+^-ATPase is expected to reduce Ca^2+^ levels inside the synaptic terminals and change I_Cl(Ca)_ I/V. However, the delocalization of MPP4 proteins in MUT rods also reduces Ca^2+^-ATPase trafficking to the synaptic terminal (Yang et al., 2007), therefore preventing the expected unbalance between Ca^2+^ influx and efflux. Overall, the similar I/V of I_Cl(Ca)_ despite a substantial reduction in both VGCC and TMEM16 channels in MUT rods may indicate that the extensive coupling between proteins lets the synapse cope with fluctuations in the rate of protein trafficking. However, the toll rod pays for this design is that defects of the VGCC complex lead to a structural and functional disorganization of their synapses.

The presence in photoreceptors of TMEM16B associated with PSD95 (Stöhr et al., 2009) raises the possibility that both TMEM16 channels contribute to calcium-activated chloride currents observed in intact photoreceptors and may play a role in regulating their synaptic transmission. The lack of TMEM16B in knockout mice for this channel does not lead to altered localization of synaptic proteins such as PSD95 (Billig et al., 2011), but conditions that alter the synaptic terminal of photoreceptors may lead also to delocalization of TMEM16B channels, such as in MPP4 knockout mice (Stöhr et al., 2009).

Our data show that calcium channels in WT photoreceptors form a protein complex with TMEM16A channels through interaction with the Ca_V_ 1.4 subunit, expanding our current view of the complexity achieved by this visual system synapse. Conditions able to cause dysfunction and/or delocalization of these channels can potentially exert their effect on calcium channels and vice-versa, as observed in the *Cacna2d4* mutant mice.

## Author Contributions

AC, IP, NB, GCD performed the experiments and analyzed data. LDS, CG, SC, GCD designed the experiments, contributed to discussion. LDS, CG, IP wrote the manuscript.

## Funding

This work was supported by the National Interest Research Project (PRIN 2010–2011 #2010599KBR_003 to CG and PRIN 2009 #20094CZ3M2_003 to GCD) of the Italian Ministry of Education (MIUR) and by the University of Pisa, Italy.

## Conflict of Interest Statement

The authors declare that the research was conducted in the absence of any commercial or financial relationships that could be construed as a potential conflict of interest.
